# Prevalence of maxillary canine impaction in skeletal Class III malocclusions compared to Class I malocclusions

**DOI:** 10.4317/jced.55478

**Published:** 2019-03-01

**Authors:** Gabriele Di Carlo, Matteo Saccucci, Valeria Luzzi, Gaetano Ierardo, Iole Vozza, Gian-Luca Sfasciotti, Antonella Polimeni

**Affiliations:** 1 Department of Oral and Maxillo-Facial Sciences, Sapienza University of Rome, Viale Regina Elena 287a, 00161 Rome, Italy

## Abstract

**Background:**

The aim of the present investigation was to evaluate if an orthodontic population of Class III malocclusion patients shows a different prevalence of maxillary canine impaction than Class I subjects.

**Material and Methods:**

Fifty-eight subjects were retrospectively selected and assigned to the Class I group (n= 32) or the Class III group (n= 26), depending on the ANB and WITS values. Lateral cephalograms were used to collect angular and linear measurements that described the skeletal and dental maxillary features of the subjects, while orthopantomography was used to assess the impaction or the correct eruption of the maxillary canines. An independent samples T-test or a Mann–Whitney U-test was used to compare the cephalometric values between the two groups, while a chi-squared test was used to evaluate the distribution of maxillary canine impaction between the two groups.

**Results:**

No statistically significant difference was found for the cephalometric variables, and the frequency of canine impaction showed no difference between the Class III and Class I subjects.

**Conclusions:**

Patients with skeletal Class III malocclusions did not show a different prevalence of canine impaction; therefore, such skeletal features cannot be used as a diagnostic aid for assessment of the risk of maxillary canine impaction.

** Key words:**Skeletal Class III, Angle Class III, maxillary canine impaction, tooth impaction.

## Introduction

The impaction of maxillary canines is the most frequent eruption pathology after that of mandibular and maxillary third molars, and it is found in almost 2% of the population (1). The treatment of an impacted canine is a complex multidisciplinary procedure that requires surgical exposure and orthodontic extrusion of the tooth; this requires accurate surgical and biomechanical planning (2,3) and a longer treatment time with respect to a treatment of a similar malocclusion without an impacted tooth (4). Many authors have studied methods to predict the risk of canine impaction, since a correct risk assessment would be of great clinical value. Besides the radiographic criteria developed by Ericson and Kurol (5) and further modified by other authors (6,7) to predict canine impaction from its position in a panoramic radiograph, some authors have studied if a patient’s dento-skeletal features could be used as a prognostic factor. For example, Mercuri *et al.* found that the facial skeletal features of patients with a palatally impacted canine are characterized by a horizontal and prognathic growth (8). Larsen *et al.* observed that the maxillary complex of patients with ectopic canines is enlarged transversally, but smaller in the sagittal and vertical dimensions (9). Similarly, Laurenziello *et al.* studied the cephalometric characteristics associated with palatally impacted canines and found that facial divergence (SN-GoMe angle) could be used to assess the risk of canine impaction in combination with the α-angle (the angle of the long axis of the maxillary canine to the midsagittal plane) and the distance of the canine cusp to the occlusal plane (10). Taken together, these are often the skeletal features of Class III malocclusions (11,12). Basdra *et al.* observed impacted canines in 9% of Class III subjects, compared to a frequency of 1.3% in Class II division 1 subjects (13).

The aim of the present investigation was to study if in an orthodontic population, skeletal Class III subjects exhibit a different prevalence of maxillary canine impaction compared to skeletal Class I subjects. The null hypothesis was that no difference exists in canine impaction prevalence between the two types of skeletal malocclusion.

## Material and Methods

The records of orthodontic patients treated at the XXXXXX from January 2008 to June 2018 were screened for the following inclusion criteria:

- Pre-pubertal age at treatment start;

- Class III malocclusion defined as an ANB angle <0° or a WITS appraisal <-3 mm;

- Class I malocclusion defined as an ANB angle comprised between 0° and 4°, or a WITS appraisal comprised between -3 mm and +1 mm.

The lateral cephalograms of the selected subjects were collected and anonymised with a numerical code. This retrospective study was conducted according to the Declaration of Helsinki, and written informed consent to participate was obtained from all patients. Tracings were performed by a single well-trained operator, and the following measurements were collected:

- ANB, the angle between the skeletal A-point, nasion, and the skeletal B-point;

- WITS, the distance between the perpendicular projections of the skeletal A-point and the skeletal B-point over the occlusal plane;

- Fh-NA, the angle between the Frankfurt plane and the nasion-skeletal A-point plane;

- Fh-PP, the angle between the Frankfurt plane and the palatal plane (anterior nasal spine [ANS] to posterior nasal spine [PNS]);

- ANS-PNS, the length of the palatal plane, i.e. the distance between the ANS and the PNS;

- U1-Fh, the angle between the Frankfurt plane and the long axis of the upper central incisor.

A panoramic radiograph and intraoral photographs were used to assess the normal eruption or the impaction of the maxillary canines. Depending on the ANB and/or WITS value, the subjects were assigned to the Class I group or the Class III group.

Error of the method

To evaluate the error of the method for cephalometric variables, the tracings of 20 randomly selected subjects were repeated after one week by the same operator. An intra-class correlation (ICC) coefficient was calculated between the two sets of measurements to evaluate the intra-operator reliability.

Statistical analysis

An independent samples T-test was used to compare the subjects’ age between the Class I and Class III groups, while a χ-square test was used to evaluate the gender distribution between the two groups. A Shapiro–Wilk normality test was used to evaluate the type of data distribution for all the cephalometric variables. An independent samples T-test or a Mann–Whitney U-test, depending on data distribution, was used to compare all the cephalometric variables between the Class I and Class III groups. A χ-square test was used to test the distribution of erupted and impacted maxillary canines between the two groups. The first type error was set as 0.05 for all the statistical tests.

## Results

From the 200 records screened, those of 58 subjects fulfilled the inclusion criteria and were selected for further analysis. According to the ANB and WITS values, 32 subjects were assigned to the Class I group and 26 were assigned to the Class III group. The two groups showed a similar gender and age distribution; therefore, they were comparable, and no significant effect from these two variables would have been observed ( Table 1).

**Table 1:**
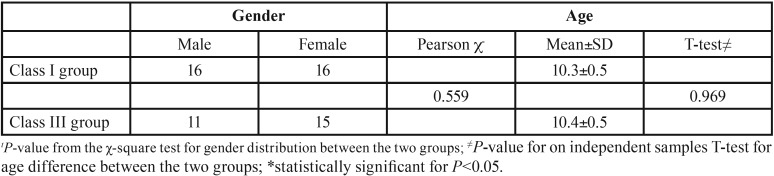
Demographic composition of the two groups.

Regarding the error of the method, the calculated ICC coefficient was excellent (> 0.85) for all the variables, revealing good intra-observer reliability of the measurements.

Descriptive statistics are reported in  Table 2. No significant differences were detected between the two groups for Fh-NA, Fh-PP, ANS-PNS, and U1-Fh. The two groups showed a significant difference regarding ANB and WITS values (*P*< 0.001), confirming that the sample was correctly sorted and that a highly different skeletal pattern was present between the groups ( Table 2). No difference in the distribution of canine impaction was present between the two groups ( Table 3); therefore, the null hypothesis was accepted.

**Table 2:**
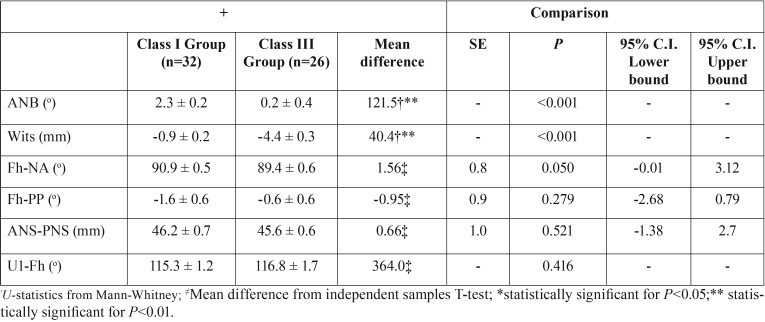
Descriptive statistics and comparison between groups for cephalometric variables.

**Table 3:**
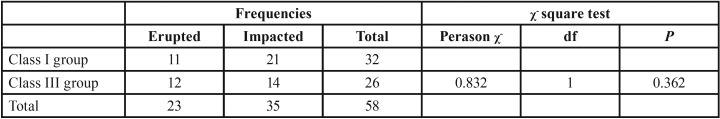
Crosstabulation and χ- square test for frequency of canine impaction between the two groups.

## Discussion

Predicting the risk of maxillary canine impaction is of great clinical importance, due to the complexity of the treatment of this eruption anomaly: the forced eruption of an impacted canine requires careful biomechanical planning(14) and the use of an orthodontic force magnitude in a physiologic range (15-17) to reduce the risks of root resorption and loss of vitality of the impacted tooth. In addition, a proper anchorage is needed, sometimes involving the use of a miniscrew (18,19), which, on the other hand, represents an additional surgical procedure with its own risks and clinical assessment, requiring also the availability of a sufficient space in a convenient location (20-22). If the risk of maxillary canine impaction is recognized at an early age, an attempt can be made to try to change the eruptive path of the canine; this involves the extraction of the deciduous canine (23,24) and rapid maxillary expansion (25,26), possibly with a device anchored onto the deciduous molars to reduce the side effects on permanent teeth (27-30).

Many authors have tried to study the dento-skeletal features that could predict an increased risk of maxillary canine impaction. Mercuri *et al.* observed that patients with impacted canines are characterized by a horizontal and prognathic growth, but they concluded that palatally displaced canines and buccally displaced canines are not associated with altered skeletal features (8). Basdra *et al.*, on the other hand, found that canine impaction was associated with Class II division 2 malocclusions in 33.5% of cases (31) and in 9% of Class III subjects (13). Sacerdoti and Baccetti found a prevalence of canine impaction in hypodivergent patients three times higher than in normal subjects, confirming an association with vertical craniofacial features (32). In addition, Larsen *et al.* found that patients with impacted canines had a significantly transversally enlarged maxilla, but sagittally and vertically smaller than subjects without impaction, suggesting the need for a three-dimensional evaluation of space for cases with ectopic canines (9).

Some authors have suggested that the association between some skeletal malocclusions and certain tooth anomalies could be due to genetic factors, rather than environmental factors (i.e., a shorter maxilla that makes it more difficult for the canine to erupt) (33). Basdra *et al.* reported that Class II division 2 malocclusion was associated with genetic-dependent tooth anomalies like agenesis of upper lateral incisors and peg-shaped incisors, in addition to impacted canines (31). Other authors have observed a correlation between maxillary retrognathia and maxillary canine-first premolar transposition (34), a dental anomaly that has been demonstrated to share a common genetic origin with palatally displaced canines (35).

Overall, the existing literature does not provide clear evidence about a possible association between craniofacial features and maxillary canine impaction. The results of the present study confirm that a Class III skeletal pattern cannot be used as a prognostic factor for the risk of development of maxillary canine impaction.

## Conclusions

No association between Class III skeletal features and maxillary canine impaction was found, confirming that such skeletal characteristics cannot be used as a diagnostic and prognostic aid in determining the risk of maxillary canine impaction.
